# Phylogenetic Relatedness of Several Agarwood-Producing Taxa (Thymelaeaceae) from Indonesia

**DOI:** 10.21315/tlsr2018.29.2.2

**Published:** 2018-07-06

**Authors:** Shiou Yih Lee, Maman Turjaman, Rozi Mohamed

**Affiliations:** 1 Forest Biotech Laboratory, Department of Forest Management, Faculty of Forestry, Universiti Putra Malaysia, 43400 UPM Serdang, Selangor, Malaysia; 2 Forest Microbiology Research Group, Forest Research and Development Centre, Forestry and Environmental Research Development and Innovation Agency (FOERDIA), Ministry of Environment and Forestry, Jalan Gunung Batu No. 5, Bogor, West Java, 16680, Indonesia

**Keywords:** *Aquilaria*, *Gyrinops*, Genetic Diversity, Internal Transcribed Spacer (ITS), *trn*L-*trn*F, *Aquilaria*, *Gyrinops*, Kepelbagaian Genetik, Penjarak Transkripsi Dalaman (ITS), *trn*L-*trn*F

## Abstract

Indonesia is home to several tree taxa that are harvested for agarwood. This highly valuable oleoresin ironically was the cause for some species to become vulnerable due to gluttonous human activity. However, information on the genetic diversity of these endangered trees is limited. In this study, 28 specimens representing eight species from two genera, *Aquilaria* and *Gyrinops*, were collected from ex-situ and in-situ populations in Indonesia. Phylogenetic analysis conducted on DNA sequences of the nuclear ribosomal internal transcribed spacer (ITS) and the *trn*L-*trn*F intergenic spacer regions, revealed that *Aquilaria* and *Gyrinops* are paraphyletic when *Aquilaria cumingiana* is excluded. The phylogenetic analysis for ITS and *trn*L-*trn*F showed capability to categorise agarwood-producing species based on their regions: East Indonesia and West Indonesia, using Wallace’s Line as the divider. In addition, we discuss challenges in species identification and taxonomy of agarwood-producing genera, and their conservation efforts in Indonesia.

## Abstract (ms)

Indonesia merupakan tempat kediaman kepada beberapa taksa pokok yang dituai untuk kayu gaharu. Oleoresin yang sangat berharga ini secara ironinya telah menyebabkan beberapa spesies pokok penghasil gaharu terancam akibat ketamakan dan kerakusan manusia. Walau bagaimanapun, maklumat mengenai kepelbagaian genetik pokok-pokok terancam ini amatlah terhad. Dalam kajian ini, sebanyak 28 spesimen yang mewakili lapan spesies dari dua genera iaitu *Aquilaria* dan *Gyrinops* telah dikumpulkan dari populasi *ex-situ* dan *in-situ* di Indonesia. Analisis filogenetik yang dijalankan pada jujukan rantau penjarak transkripsi dalaman (ITS) ribosom nuklear dan rantau penjarak intergenik *trn*L-*trn*F telah menunjukkan bahawa *Aquilaria* dan *Gyrinops* adalah parafiletik apabila *Aquilaria cumingiana* dikecualikan. Analisis filogenetik untuk ITS dan *trn*L-*trn*F juga menunjukkan keupayaan untuk mengkategorikan spesies pokok penghasil gaharu berdasarkan kawasan masing-masing: Indonesia Timur dan Indonesia Barat, dengan menggunakan Garisan Wallace sebagai garisan pembahagi. Tambahan, kami juga turut membincangkan cabaran dalam pengenalpastian spesies dan taksonomi genera penghasil gaharu serta usaha dalam pemuliharaannya di Indonesia.

## INTRODUCTION

**Figure 1:**
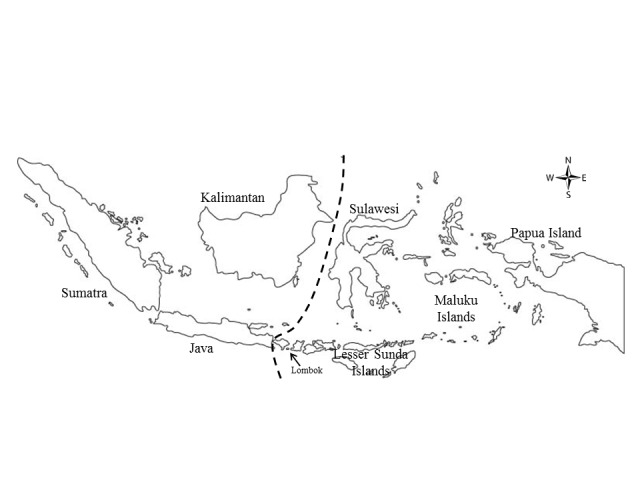
Geographical separation for Indonesia based on the Wallace Line. East Indonesia covers the area on the right side of the separating line, which includes Sulawesi, Lesser Sunda Islands, Maluku Islands and Papua Island, while West Indonesia is on the left side and it includes Sumatra, Java and Kalimantan.

Agarwood is one of the non-timber forest products (NTFPs) collected from the wild since several decades ago (Edy Komar *et al.* 2014). In more recent time, it receives much attention as one of the most important NTFPs with unbelievably high prices broadcasted for its premium quality (Karlinasari *et al.* 2015). In Indonesia, agarwood has been reportedly produced by seven genera: *Aetoxylon*, *Aquilaria*, *Enkleia*, *Gonystylus*, *Gyrinops*, *Phaleria* and *Wikstroemia* (Roemantyo & Partomihardjo 2010, Leksono & Widyatmoko 2012, Edy Komar *et al.* 2014). *Aquilaria* and *Gyrinops* are the major genera with a total of six and seven species, respectively (Table 1). *Aquilaria* is mostly distributed in the western part of Indonesia, while *Gyrinops* dominates the eastern part (Fig. 1). Plantations of *Aquilaria* are widespread in Indonesia when compared to *Gyrinops*. *Aquilaria* is a preferred agarwood source in large-scale plantations compared to *Gyrinops* because *Gyrinops* is slow-growing. Wild populations of *Gyrinops* are still present in the eastern part of Indonesia but information on this species is hard to obtain due to the difficulty to access the natural populations and underdeveloped civilisation, therefore it is less studied. Species identification is difficult as their main differences are contributed by their reproductive structures, mainly flower and fruit (Susilo *et al.* 2014). Hou (1960) separated the two genera based on a single morphological characteristic, the number of stamens. *Gyrinops* has a series of five stamens, while in *Aquilaria* the number doubled (Eurlings & Gravendeel 2005). Consequently, it would be challenging to identify the trees with confidence, when relying on vegetative characteristics alone, while identifying the right species of agarwood-producing tree is important as different species produce different agarwood quality (Rimbawanto & Widyatmoko 2011). Agarwood employs a high price tag and thus receives substantial publicity from the media and marketing strategists. This drives local people in Indonesia to venture into agarwood tree cultivation. In order to come out with an effective breeding system for these species, the identification of the species and gene pool is an essential step.

Molecular approaches using DNA gene markers have been widely applied to identify the genetic variation of plant species and were first reported in agarwood-producing tree species in 2005. Several genes such as the intergenic spacer region *psb*C-*trn*S and the nuclear ribosomal internal transcribed spacer region 1 (ITS1) (Ito & Honda 2005), internal transcribed spacer region (ITS) (Lee & Mohamed 2016a) and the intergenic spacer region *trn*L-*trn*F (Eurlings & Gravendeel 2005, Lee & Mohamed 2016a) have been utilised on various *Aquilaria* and *Gyrinops* species. Although DNA-based identification is feasible, verification on species identity via conventional techniques maybe hindered when the reproductive parts are absent. This is exacerbated by the lack of reliable sequences in public DNA databases. Expanding the database with sequences from authentic sources may help in providing a fast and efficient identification support. In Indonesia, information on the genetic diversity of both *Aquilaria* and *Gyrinops* species is non-existence except for one published report on the genetic variations of cultivated species using Amplified Fragment Length Polymorphism (AFLP) markers (Toruan-Mathius *et al.* 2009).

In this study, we utilised the *trn*L-*trn*F and ITS regions to determine the genetic diversity and phylogenetic affinities among eight agarwood-producing species from Indonesia. We identify the challenges in species identification and conservation of agarwood-producing species in Indonesia. The information we produced can be used in assisting conservation efforts of these threatened species in Indonesia.

## MATERIALS AND METHODS

### Samples Collection

Plant samples were collected from 31 individuals representing nine species. Whenever possible, samples in the form of fresh leaves were collected and used directly in genomic DNA extraction. Otherwise, they were oven-dried at 60°C overnight before being transported back to the laboratory. Samples were provided by the Forestry and Environmental Research Development and Innovation Agency (FOERDIA), Bogor, Indonesia (Table 2).

### Molecular Methods

Genomic DNA was extracted using the DNeasy Plant Minikit (Qiagen, Germany). The quantity and quality were determined by spectrophotometry (Nanophotometer, IMPLEN, USA). Genomic DNA extracted from leaf samples were considered yielding DNA of good quality with A_260_/A_280_ ratio between 1.700 and 1.900 (Sambrook & Russel 2001). PCR amplification of the *trn*L-*trn*F intergenic spacer region was carried-out using primer E: 5′-GGT TCA AGT CCC TCT ATC CC-3′, and primer F: 5′-ATT TGA ACT GGT GAC ACG AG-3′ (Taberlet *et al.* 1991), while the nuclear ribosomal ITS region was amplified using primer ITS-p5: 5′-CCT TAT CAY TTA GAG GAA GGA G3′, and ITS-u4: 5′-RGT TTC TTT TCC TCC GCT TA-3′ (Cheng *et al.* 2015). PCR reaction was prepared in a 25 μl volume containing 12.5 μl of PCRBioTaq Mix Red (PCR Biosystems, UK), 0.4 μM of each primer, 5 – 25 ng of genomic DNA. PCR amplification was carried-out in a SpeedCycler^2^ (Analytik Jena, Germany) as follows: denaturation at 95°C for 1 min, 40 cycles of 95°C for 15 s, 55°C for 15 s, and 72°C for 15 s, and a final extension of 72°C for 3 min. PCR products were sent for direct sequencing at First BASE Laboratories Sdn Bhd, Selangor, Malaysia, on an ABI PRISM 3730xl Genetic Analyzer (Applied Biosystems, USA).

### Phylogenetic Analyses

DNA sequence contigs were edited using the program Gene Runner version 3.05 (Hastings Software Inc., USA) with subsequent manual adjustments. Sequences obtained from this study were deposited into the GenBank (Table 2). A local DNA sequence database was set-up by mining through the GenBank for related known sequences. All sequences were aligned using the program Clustal W implemented in the software MEGA 6 (Tamura *et al.* 2013) and intra-specific genetic variation was analysed for tree species with more than one sample. DNA substitution models which are suitable for the two genes were assessed using the “find best DNA/Protein Models (ML)” function embedded in the software MEGA 6 by implementing the maximum likelihood statistical method to test the goodness of fit to several models of evolution. According to the estimated values of all parameters for each model, the model best fitting to the dataset from the sequence *trn*L-*trn*F was Hasegawa-Kishino-Yano (HKY) model, while the dataset from sequence ITS was Kimura two-parameter (K2P) model and invariable sites (+I) model (=K2P+I). Phylogenetic tree was constructed based on the maximum likelihood criteria implemented in MEGA 6. Inter- and intraspecific pairwise distances was calculated based on Kimura two-parameter model (Kimura 1980), and all positions containing gaps and missing data were included for analysis. Clade supports were calculated based on 1000 bootstrap resamplings. *Gonystylus bancanus, G. macrophyllus* and *Phaleria macrocarpa* were included as out-groups in the *trn*L-*trn*F tree, while for the ITS tree only *Gonystylus bancanus* and *Phaleria macrocarpa* were included as out-groups. These species were selected as out-groups because they are closely-related to *Aquilaria* and *Gyrinops* (Lee & Mohamed 2016b). The two genes were not analysed together as they are from two different inheritance systems.

## RESULTS

### DNA Sequences

A total of 62 sequences were obtained from this study, however intraspecific variation was not observed among individuals from the same population. Therefore, only 24 sequences (12 from each gene) were selected from the different populations as representative sequences and deposited in the GenBank (Table 2). When comparing the *trn*L-*trn*F sequences from all the eight species studied, the lengths of the sequences were between 496 bp to 500 bp. No genetic variation was observed within each species. The final alignment of the *trn*L-*trn*F sequences had a total of 501 bp, with four polymorphic sites and six indels, and four parsimoniously informative sites. The interspecific pairwise distance was greatest between *A. microcarpa* and *G. caudata* (0.0081), and no genetic differentiation was observed between *A. beccariana* and *A. malaccensis*, and between *A. cumingiana*, *G. ledermannii*, *G. moluccana*, and *G. versteegii* (Table 3(a)).

The ITS sequences on the other hand were of the same length, 683 bp, for all species except *G. versteegii* from Lombok (682 bp). No genetic variation was observed within each species, except for *G. versteegii* (0.0316) from four different populations (data not shown). The final alignment had a total length of 687 bp, with 72 polymorphic sites and seven indels, and had six parsimoniously informative sites. The interspecific pairwise distances for ITS sequences ranged from 0.0286 to 0.0551, with only one species pair, *A. beccariana* and *A. malaccensis,* showing no genetic differences (Table 3(b)).

### Phylogenetic Analysis

**Figure 2:**
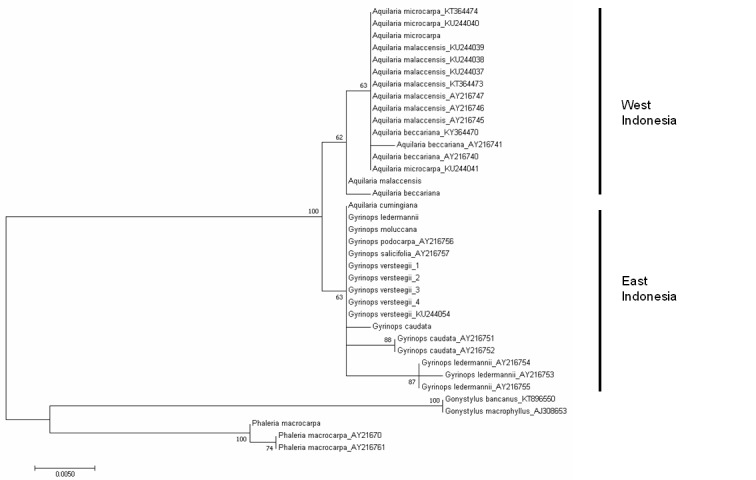
Maximum likelihood tree constructed using the intergenic spacer region *trn*L-*trn*F sequences obtained from this study and from the NCBI GenBank. The origin of each species used in this study is listed in Table 2. *G. versteegii* populations are annotated to their respective origins: (1) Lombok, (2) Lesser Sunda Islands, (3) Papua Island, and (4) Maluku Islands. Sequences with accession numbers following species names are from the NCBI GenBank and as listed in Table 4. *Gonystylus bancanus, G. macrophyllus* and *Phaleria macrocarpa* is treated as out-group. Bootstrap values (1000 replicates) are shown at the branches.

**Figure 3:**
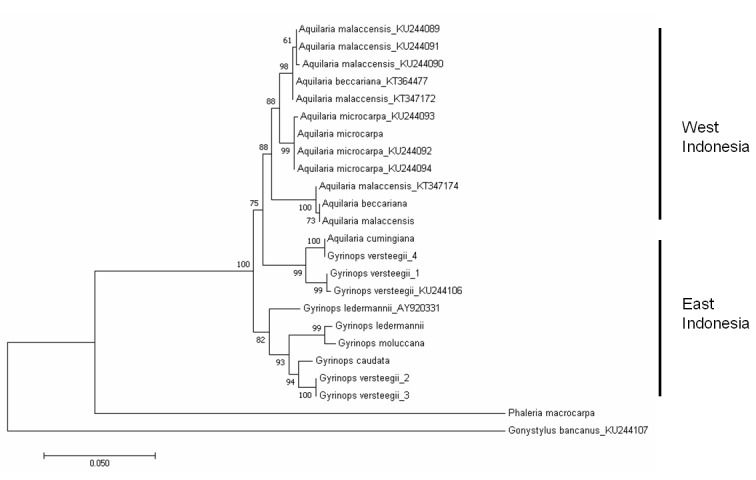
Maximum likelihood tree constructed using the nuclear ribosomal ITS sequences obtained from this study and from the NCBI GenBank. The origin of each species used in this study is listed in Table 2. *G. versteegii* populations are annotated to their respective origins: (1) Lombok, (2) Lesser Sunda Islands, (3) Papua Island, and (4) Maluku Islands. Sequences with accession numbers following species names are from the NCBI GenBank and as listed in Table 4. *Gonystylus bancanus* and *Phaleria macrocarpa* is treated as out-group. Bootstrap values (1000 replicates) are shown at the branches.

The *trn*L-*trn*F tree was constructed using DNA sequences obtained in this study, as well as sequences downloaded from GenBank (Table 4). The tree formed two clusters, mainly separating *Aquilaria* and *Gyrinops*, with moderate bootstrap support (63% and 87%) (Fig. 2), except for *A. cumingiana*. The ITS tree was constructed using only sequences obtained in this study. Generally, *Gyrinops* and *Aquilaria* were clustered separately, apart from *A. cumingiana*, which was clustered together with *Gyrinops* (Fig. 3). The branching of *Aquilaria* and *Gyrinops* also showed moderate (66%) bootstrap support.

## DISCUSSION

### *Aquilaria* and *Gyrinops* are Paraphyletic

This is the first report on molecular phylogeny of *Aquilaria* and *Gyrinops* species distributed in Indonesia. If *A. cumingiana* is excluded, the phylogenetic analysis using the *trn*L-*trn*F sequences showed *Aquilaria* and *Gyrinops* are paraphyletic, similar to Eurlings and Gravendeel (2005). Furthermore, based on the Wallace Line, the general clustering in the phylogenetic tree from *trn*L-*trn*F and ITS sequences (Fig. 2) correctly placed the agarwood-producing species into their respective regions, *i.e.* East Indonesia and West Indonesia. The eastern region comprises of Sulawesi, Lesser Sunda Islands, Maluku Islands and Papua Island, and the western region comprises of Sumatra, Java and Kalimantan (Fig. 1). In this study, the agarwood-producing species that clustered in the eastern region were all the *Gyrinops* species except for *A. cumingiana*, while the rest of the *Aquilaria* species clustered under the western region (Fig. 2 & 3). Unlike the *trn*L-*trn*F tree, the ITS tree showed *Gyrinops* to be ancestral to *Aquilaria* (Fig. 3). The ITS region has been reported as a useful tool in plant phylogenetic studies (Baldwin *et al.* 1995, Alvarez & Wendel 2003). Its biparental inheritance characteristic can be utilised to differentiate their respective populations. Application of the ITS region for genetic diversity studies has been proven successful in *Aquilaria*. Several studies on *A. sinensis* showed that ITS was able to distinguish populations from various provinces in China, and a recent finding on that showed ITS was able to tell apart populations of different countries (Lee *et al.* 2017). Genetic isolation and genetic fragmentation due to urbanisation at early times led to high genetic variations among the *A. sinensis* at various locations (Shen *et al.* 2008, Niu *et al.* 2010). Similarly, ITS was able to discern *G. versteegii* populations from three provincial islands: 1) Lombok, 2) Maluku, and 3) Lesser Sunda and Papua. These populations are distantly separated and genetically isolated by the sea. Due to biparental inheritance in the nuclear ribosomal ITS region, the *G. versteegii* populations did not cluster together under the same branch. As *Gyrinops* is closely related to *Aquilaria*, we conclude that the high intraspecific genetic variation in the ITS sequence among *G. versteegii* caused populations from Lombok and Maluku Islands to cluster with *Aquilaria* and not with those from Lesser Sunda and Papua.

### Taxonomy Challenges between *Aquilaria*, *Gyrinops* and *Gyrinopsis*

Interestingly, based on both sequences, *A. cumingiana* appeared to nestle under the *Gyrinops* clade. We cannot rule out the possibility that *A. cumingiana* may be closer to *Gyrinops* as it was previously identified as *Gyrinopsis cumingiana* (The Plant List 2013). The genus *Gyrinopsis* was first reported by Decaisne (1843) with *G. cumingiana* found in the Philippines as the type specimen. Later, Ridley (1901) addressed it as *A. cumingiana* when he reported the discovery of *A. hirta*, which is highly similar to *A. cumingiana*. The latter has a long perianth tube and fruit that develops by breaking the side of the perianth tube. This characteristic is also shared by *A. hirta*, only that it is puberulous on the leaf abaxial and fruit surface, which *A. cumingiana* is lacking. The debate to retain the genus *Gyrinopsis* was raised by Quisumbing (1946) when he reported a critical study on the Philippine species under the Aquilarieae tribe. Quisumbing (1946) pointed out that the distinct perianth tube of *Gyrinopsis* is a major distinguishing feature that should not be ignored. Eventually all *Gyrinopsis* species, except *G. cumingiana*, were reported as endemic to the Philippines. The genus *Gyrinopsis* was then officially synonymised with *Aquilaria* because they shared the same number of stamens (10 stamens); while *Gyrinops* only had five stamens (Hou 1960). From our observations in the field, we found that the two genera *Aquilaria* and *Gyrinops* can be separated by a distinct characteristic - the colour of the mature fruit. *Aquilaria* often has green fruits, while those of *Gyrinops* are orange or yellowish in colour. Surprisingly, *A. cumingiana* has orange to brownish mature fruit instead of green mature fruit (http://www.tropicos.org/Name/50314049). This may lead to the proposal that *A. cumingiana* be retained as *Gyrinopsis* as it has 10 stamens like *Aquilaria*, and orange fruits like *Gyrinops*. In addition, unlike the other two genera, *Gyrinopsis* has a long perianth tube. Other *Aquilaria* species that has orange mature fruit and 10 stamens is *A. filaria* (formerly known as *Pittosporum filaria*) (http://www.tropicos.org/Name/50314819). However, the colour of a mature fruit is seldom taken as a main key in plant taxonomy. Yet to differentiate between *Aquilaria* and *Gyrinops* using a single characteristic, which is the number of stamens, can as well be disputed (Eurlings & Gravendeel 2005).

### Challenges in Species Identification and Conservation of Agarwood-Producing Species In Indonesia

According to the Tropicos (http://www.tropicos.org), an established online botanical database maintained by the Missouri Botanical Garden, there are 13 agarwood-producing species from two genera being distributed over six geographical locations in Indonesia (Table 1). For the past several years, FORDA has conducted extensive field explorations and observed occurrence of other species in several islands such as Sulawesi, Lesser Sunda and Maluku. However, because the taxonomy of *Aquilaria* of Indonesian origin has not been revised for a long time, the identity of these species and the status of previously reported ones could not be ascertained. Among the main islands in Indonesia, only Java does not have a native species although popular plantation species like *A. malaccensis*, *A. microcarpa* and *G. versteegii* are currently being introduced from other parts of Indonesia into Java. Being the country with the richest diversity of agarwood-producing species, we found that there is a limitation in performing species identification even when using DNA as evidence. Generic delimitation between the genera in the Aquilarieae tribe is based on the number of stamens. In our opinion, it should not be emphasised as the sole characteristic for supporting genus level. Quisumbing (1946) suggested that stamen numbers be regarded as one of the distinguishing features. There are other characteristics such as the calyx lobes, perianth tube and fruit shape, that can be used as major features to differentiate species. In our study, a few species were inseparable by DNA evidence. For example, *A. malaccensis* and *A. beccariana* can be clearly distinguished from the distinct structure of their calyx lobes (the former is often reflexed and the latter is cylindrical), but DNA sequence was not able to resolve their identities. The two species are highly identical in vegetative morphology, which explains why they can be mis-identified when the fruits are not available. Given their high similarity in morphological and ecological aspects, we may consider them as a single evolutionary unit. In another case concerning *G. ledermannii* and *G. moluccana*, both phylogenetic trees were not able to separate them. While we suggest this could be due to the slow molecular evolution rate in the chloroplast DNA, they could be also experiencing shared ancestral polymorphism. Similar finding was also reported in *Araucaria* species (Gaudeul *et al.* 2014). While molecular identification using DNA sequences is a potential supporting tool in species identification, it was not consistent in several genera. Other DNA-based method such as amplified fragment length polymorphisms (AFLP) has been suggested to tackle this problem (Després *et al.* 2003, Gaudeul *et al.* 2012). However, for agarwood, which is often traded in wood or wood products, the only other option is through wood anatomy. Its major limitation is the technique can only disclose down to the genus level (Gasson 2011). The current practice of identifying agarwood source of origin through wood anatomy is based on the presence of included phloem (Kim & Park 2011), which is present only in members of the Aquilarieae tribe under the family Thymelaeaceae, and is absent from the other tribes (Asdar 2007). A preliminary study on the anatomy structure of five different agarwood-producing species (*A. beccariana*, *A. malaccensis*, *A. microcarpa*, *A. cumingiana (*previously known as *Gyrinopsis cumingiana)* and *G. versteegii*,) found that the genera under the Aquilarieae tribe could be distinguished using other anatomical features (Andianto 2010). For example, *Aquilaria*’s vessels are arranged in radial multiples of two to three common cells, while *Gyrinops* and *Gyrinopsis* have two to eight common cells. *Gyrinops* and *Gyrinopsis* have differences in ray width, *G. versteegii* has uniseriate width, while *A. cumingiana* has uni-to bi-seriate width. This shows that *A. cumingiana* is more similar in its anatomical feature to *G. versteegii* than *Aquilaria* species. Our results in the form of phylogenetic trees (Figs. 3 and 4) also displayed similar findings. Should there be any efforts to revise the species names, we suggest one of the followings to be taken into consideration: 1) the genus *Gyrinopsis* to be retained due to its distinct anatomical feature in ray width, 2) to merge *Gyrinopsis* species having 10 stamens with *Gyrinops* instead of *Aquilaria* if they share identical number of vessels in radial multiples or having a yellow/orange mature fruit, or 3) to consolidate all the three genera under *Aquilaria* as they all possess included phloem. We also suggest including anatomical features as another reference point in taxonomical classification, at genus level the least, due to the diversity of these agarwood-producing species.

## CONCLUSION

The chloroplast *trn*L-*trn*F sequences displayed capability in discerning the many species of the agarwood-producing trees and clustered them to their respective geographical origin. However, the moderate bootstrap support level reflects the limitation of the *trn*L-*trn*F region in differentiating each species with confidence. On the other hand, the ITS region may not be a suitable gene to characterise both genera due to high genetic variation observed within the same species. The utilisation of ITS region to identify wood samples may be promising under the condition that large sampling area from various populations were carried out to include all possibilities in sequence variation. Considering the threatened status of several species in their natural population, effort in sampling may be difficult and sensitive. Therefore, this study may serve as a reference in establishing a systematic conservation program in preserving the genetic diversity of these agarwood-producing species in Indonesia.

## Figures and Tables

**Table 1:** An updated list of native agarwood-producing tree species distributed in Indonesia.

Agarwood-producing species	Geographical location^a^
*A. beccariana*	Kalimantan, Sumatra
*A. cumingiana*	Kalimantan, Maluku Islands, Sulawesi^b^
*A. filaria*	Lesser Sunda Islands^b^, Maluku Islands, Papua Island
*A. hirta*	Kalimantan^b^, Sumatra
*A. malaccensis*	Kalimantan, Sumatra
*A. microcarpa*	Kalimantan, Sumatra
*G. caudata*	Papua Island
*G. decipiens*	Sulawesi
*G. ledermannii*	Maluku Islands^b^, Papua Island
*G. moluccana*	Maluku Island
*G. podocarpus*	Papua Island
*G. salicifolia*	Papua Island
*G. versteegii*	Sulawesi, Lesser Sunda Islands, Maluku Islands^b^, Papua Island

a *Source*: Tropicos, botanical information system at the Missouri Botanical Garden (http://www.tropicos.org)

b Reported here through this study

*Note:* A = *Aquilaria*; G = *Gyrinops*

**Table 2:** Origins and sources of the agarwood-producing species used in this study. The *trn*L-*trn*F and ITS sequences obtained from this study are deposited in the GenBank.

Species	Individuals	Origin	Source	Voucher specimen	GenBank accession no.	

					*trn*L-*trn*F	ITS
*A. beccariana*	2	Kalimantan	FOERDIA^a^	FBL04001	KT726319	KT779116
*A. cumingiana*	2	Maluku Islands	BURU^c^	MTJ0006	KT726320	KT779117
*A. malaccensis*	4	Sumatra	BBG^b^	MTJ0001	KT726321	KT347174
*A. microcarpa*	3	Kalimantan	FOERDIA^a^	FBL01018	KT726322	KT779118
*G. caudata*	4	West Papua	BBG^b^	MTJ0002	KT726323	KT779119
*G. ledermannii*	1	Maluku Islands	BURU^c^	MTJ0007	KT726324	KT779120
*G. moluccana*	2	Maluku Islands	BURU^c^	MTJ0008	KT726325	KT779121
*G. versteegii*	3	Lombok Island	FOERDIA^a^	FBL01027	KT726326	KT779122
	2	Maluku Island	BURU^c^	MTJ0009	KT726329	KT779123
	3	Lesser Sunda Islands	BBG^b^	MTJ0003	KT26327	KT779124
	2	Papua Island	BBG^b^	MTJ0004	KT726328	KT779125
*Phaleria macrocarpa*	2	Papua Island	BBG^b^	MTJ0005	KT726330	KT779126

a ex-situ trees planted at the arboretum of the Forestry and Environmental Research Development and Innovation Agency (FOERDIA), Bogor, Indonesia

b ex-situ trees planted at the Bogor Botanical Garden (BBG), Bogor, Indonesia

c in-situ trees planted at Buru Island, Maluku Islands, Indonesia (collected and identified by Dr. M. Turjaman)

*Note:* A = *Aquilaria*; G = *Gyrinops*

**Table 3:** Interspecific pairwise distances of sequences between several agarwood-producing species used in this study.

(a) *trn*L-*trn*F	*A. beccariana*	*A. cumingiana*	*A. malaccensis*	*A. microcarpa*	*G. caudata*	*G. ledermannii*	*G. moluccana*
*A. beccariana*	-	-	-	-	-	-	-
*A. cumingiana*	0.0040	-	-	-	-	-	-
*A. malaccensis*	0.0000	0.0040	-	-	-	-	-
*A. microcarpa*	0.0020	0.0061	0.0020	-	-	-	-
*G. caudata*	0.0061	0.0020	0.0061	0.0081	-	-	-
*G. ledermannii*	0.0040	0.0000	0.0040	0.0061	0.0020	-	-
*G. moluccana*	0.0040	0.0000	0.0041	0.0061	0.0020	0.0000	-
*G. versteegii*	0.0040	0.0000	0.0040	0.0061	0.0020	0.0000	0.0000

(b) ITS	*A. beccariana*	*A. cumingiana*	*A. malaccensis*	*A. microcarpa*	*G. caudata*	*G. ledermannii*	*G. moluccana*

*A. beccariana*	-	-	-	-	-	-	-
*A. cumingiana*	0.0456	-	-	-	-	-	-
*A. malaccensis*	0.0000	0.0457	-	-	-	-	-
*A. microcarpa*	0.0300	0.0393	0.0301	-	-	-	-
*G. caudata*	0.0502	0.0504	0.0503	0.0423	-	-	-
*G. ledermannii*	0.0534	0.0472	0.0535	0.0486	0.0284	-	-
*G. moluccana*	0.0550	0.0425	0.0551	0.0502	0.0300	0.0074	-
*G. versteegii*	0.0484	0.0286	0.0485	0.0414	0.0344	0.0394	0.0382

Note: A. = Aquilaria; G. = Gyrinops

**Table 4:** GenBank references of the selected species included in phylogenetic tree construction.

Region	Species	GenBank Acession No.	Reference
*trn*L*-trn*F	*A. beccariana*	AY216740, AY216741	Eurlings & Gravendeel (2005)
		KT364473	Lee *et al.* (2016)
	*A. malaccensis*	AY216745, AY216746, AY216747	Eurlings & Gravendeel (2005)
		KT364473	Lee & Mohamed (2016a,b)
		KU244037, KU244038, KU244039	Lee *et al.* (2016)
	*A. microcarpa*	KT364474	Lee & Mohamed (2016a,b)
		KU244040, KU244041	Lee *et al.* (2016)
	*G. caudata*	AY216751, AY216752	Eurlings & Gravendeel (2005)
	*G. ledermannii*	AY216753, AY216754, AY216755	Eurlings & Gravendeel (2005)
	*G. podocarpa*	AY216756	Eurlings & Gravendeel (2005)
	*G. salicifolia*	AY216757	Eurlings & Gravendeel (2005)
	*G. versteegii*	KU244054	Lee *et al.* (2016)
	*Phaleria macrocarpa*	AY216760, AY216761	Eurlings & Gravendeel (2005)
	*Gonystylus bancanus*	KT896550	Lee & Mohamed (2016a,b)
	*Gonystylus macrophyllus*	AJ308653	Van der Bank *et al.* (2002)

ITS	*A. beccariana*	KT364477	Lee & Mohamed (2016a,b)
	*A. malaccensis*	KT364480	Lee & Mohamed (2016a,b)
		KU244089, KU244090, KU244091	Lee *et al.* (2016)
		KT347172, KT347174,	Lee *et al.* (2017)
	*A. microcarpa*	KT264481	Lee & Mohamed (2016a,b)
		KU244092, KU244093, KU244094	Lee *et al.* (2016)
	*G. ledermannii*	AY920331	Kiet *et al.* (2005)
	*G. versteegii*	KU244106	Lee *et al.* (2016)
	*Gonystylus bancanus*	KU244107	Lee *et al.* (2016)
